# P02.28. Non-verbal communication of compassion: feasibility of measuring psychophysiological effects of blind exposure

**DOI:** 10.1186/1472-6882-12-S1-P84

**Published:** 2012-06-12

**Authors:** K Kemper, H Shaltout

**Affiliations:** 1 Wake Forest University Health Sciences, Winston-Salem, USA

## Purpose

Calm, compassionate clinicians comfort others. This preliminary study was designed to a) test the feasibility of two strategies for maintaining subject blinding to non-verbal communication of compassion (NVCC), and b) determine whether blinded subjects would experience psychophysiologic effects from NVCC.

## Methods

Subjects were healthy volunteers who were told the study was evaluating the effect of time and touch on the autonomic nervous system. The practitioner had more than 10 years experience with lovingkindness meditation (LKM), a form of NVCC. Subjects completed 10-point visual analog scales (VAS) for stress, relaxation and peacefulness before and after LKM. To assess physiologic effects, practitioners and subjects wore cardiorespiratory monitors to assess respiratory rate (RR), heart rate (HR) and heart rate variability (HRV) throughout the 4 10-minute study periods: Baseline (both practitioner and subjects read neutral material); non-tactile-LKM (subjects read while the practitioner practiced LKM while pretending to read); tactile-LKM (subjects rested while the practitioner practiced LKM while lightly touching the S on arms, shoulders, hands, feet, and legs); Post-Intervention Rest (subjects rested; the practitioner read). To assess blinding, subjects were asked what the practitioner was doing during each period.

## Results

Subjects’ mean age was 43.6 years; all were women. Blinding was maintained and the practitioner was able to maintain meditation for both tactile and non-tactile LKM interventions as reflected in significantly reduced RR. Despite blinding, subjects’ VAS scores improved from baseline to post-intervention for stress, relaxation and peacefulness (p<0.05 for all comparisons). Subjects also had significant reductions in RR (p<0.0001) and improved HRV (p<0.05) with both tactile and non-tactile LKM.

## Conclusion

It is possible to test the effects of LKM with two blinding strategies; even with blinding, subjects reportedimprovements in well-being, reflected in objective physiologic measures of autonomic activity. Extending compassion is not only good care; it may also be good medicine.

